# Pyroresistive Properties of Composites Based on HDPE and Carbon Fillers

**DOI:** 10.3390/polym15092105

**Published:** 2023-04-28

**Authors:** Yevgen Mamunya, Oleksii Maruzhenko, Roman Kolisnyk, Maksym Iurzhenko, Andrii Pylypenko, Olha Masiuchok, Marcin Godzierz, Igor Krivtsun, Barbara Trzebicka, Sébastien Pruvost

**Affiliations:** 1 Institute of Macromolecular Chemistry of NAS of Ukraine, Kharkovskoe Chaussee 48, 02160 Kyiv, Ukraine; 2 E.O. Paton Electric Welding Institute of NAS of Ukraine, Kazymyra Malevycha 11, 03680 Kyiv, Ukraine; 3 International Polish-Ukrainian Research Laboratory ADPOLCOM; 4 Department of Electrical and Computer Engineering, University of Minnesota Twin Cities, Union St SE 200, Minneapolis, MN 55455, USA; 5 Centre of Polymer and Carbon Materials, Polish Academy of Sciences, 34 ul. M. Curie-Skłodowskiej, 41-819 Zabrze, Poland; 6 Université de Lyon, CNRS, Université Claude Bernard Lyon 1, INSA Lyon, Université Jean Monnet, UMR 5223, Ingénierie des Matériaux Polymères, CEDEX, F-69621 Villeurbanne, France

**Keywords:** electrical/thermal properties, compression moulding, polymer matrix composites (PMCs), pyroresistivity

## Abstract

Electrothermal processes were studied in pyroresistive composites based on high-density polyethylene (HDPE) containing 8 vol.% carbon black (CB), 8 vol.% carbon fibers (CF), and their mixture 4 vol.% CB + 4 vol.% CF. It is shown that the kinetic heating curves of composites are well described by an exponential dependence with a certain heating rate constant *k* for each type of composite. After a short heating time, the equilibrium temperature *T_e_* is reached in the sample. When the applied voltage exceeds a certain value, the *T_e_* value decreases due to the presence of the positive temperature coefficient of resistance (PTC) effect. Due to the PTC effect, the composites exhibit a self-regulating effect relative to the *T_e_*. Relations between the applied voltage, electric power, and equilibrium temperature are found, the *T_e_* value depends on the applied voltage according to the quadratic law whereas there is a linear relationship between the *T_e_* and electric power. A possible application of such pyroresistive composites is resistance welding of plastics using a heating element (HE) made of a pyroresistive material. The use of HDPE-CB composite to create HE for resistance welding is demonstrated and the welded joint of HDPE parts obtained using HE is shown.

## 1. Introduction

Conductive polymer composites (CPCs) attract a lot of attention mainly due to their numerous applications as antistatic, electromagnetic interference shielding materials, conductive films, conductive coatings, and various sensors based on piezoresistive and chemoresistive effects. One of the possible uses is materials for electric heaters, which are classified as materials with a pyroresistive effect [1,2,3,4,5].

Polymer composites with a high level of electrical conductivity can be used for electrical heating materials, that is, devices that are electrical resistors that convert electrical energy into thermal [2,6,7]. Metallic electrical heating devices have some disadvantages, such as oxidative corrosion and high production costs, and difficulties in manufacturing complex-shaped heaters. In turn, polymer composites containing carbon fillers [8] have such advantages as lightness and corrosion resistance, they are easy to process, and have lower costs for the production of heaters. However, it should also be noted an important drawback of such composites is rapid aging [9].

High conductivity of composites generally requires high filler concentrations. However, improving the electrical conductivity by introducing more conductive filler leads to the degradation of the mechanical properties of the composite. Therefore, much attention has been focused on the processing of conductive composites with low filler content by using 1D/2D fillers [10,11], hybrid fillers [12], and localization of fillers [13,14,15,16,17]. Good examples of the latter are segregated systems in which the conductive filler is located in certain areas inside the polymer matrix, forming a conductive framework, in contrast to the randomly distributed filler particles in conventional systems [18,19,20]. In this case, the local concentration of the filler in the walls of the framework is much higher than the average, related to the entire volume of the polymer matrix, which ensures high electrical conductivity at a low filler content.

When forming a CPC for a specific application, it is necessary to determine the type of polymer matrix, filler, and its distribution. In [21,22,23], the influence of the type of matrix on the pyroresistive properties of composites was established, and the carbon filler CNTs (carbon nanotubes) were compared with metalized glass spheres [21].

It was shown that CNT is a more promising filler for the practical application of composites. The effect of carbon fillers and their geometry on the heating properties of composites was also studied in [24]. In addition, synergistic effects were obtained when using hybrid carbon fillers [25,26,27]. As mentioned above, the distribution of the filler has a significant influence on the electrophysical properties. In the case of cellulose paper impregnated with CNT [28,29], the distribution can be considered pseudo-segregated, since the filler is located on the surface of the polymer fibers, which leads to high electrical conductivity and a low percolation threshold. In another work [30], nearly transparent two-layer composites based on polydimethylsiloxane and CNT were obtained.

The above-described CPCs have potentially wide applications as safety batteries, resettable fuses, temperature sensors, and self-regulating heating devices with the prospect of use in electronics, heaters, aircraft construction, etc. [1]. For example, it was proposed [31] to use a fabric with copper nanofibers covered with two layers of polymers, which gives the weaving highly elastic properties with the necessary level of conductivity for use in smart clothing with the ability to adjust the temperature. Pyroresistive materials have the potential for use in aircraft design as a de-icing layer [32]. A two-layer de-icing material consisting of thermoplastic polyurethane and CNT has also been proposed, in which the presence of two layers makes it possible to form a highly elastic composite with uniform heating [33]. The possible use of CPC based on polypropylene filled with carbon black as heating elements was also analyzed in [34]. In general, such materials are promising since they have a relatively low consumption of electrical energy [29], a wide temperature range, up to 200–250 °C [27,35], and good reproducibility for heating-cooling cycles [36]. The composites can fully utilize the respective advantages of each component.

The pyroresistive composites mentioned above were produced based on various types of polymers; however, there are no data in the literature on the creation of pyroresistive materials based on polyethylene (with the exception of [23,37], where ultra-high-molecular-weight polyethylene (UHMWPE)-CNT films and composites based on HDPE-TPU blend were studied). Meanwhile, the use of pyroresistive materials based on PE, the most widely used polymer in the industry, is very promising for use as a heating element in the welding of polyethylene pipes and other PE products [38]. The heating element, which melts the parts to be welded in the weld zone and remains there after cooling, ensures the strength of the joint at the level of the parts to be joined if it is made based on the same polymer as the parts to be welded. From this point of view, the study of pyroresistive materials based on PE is very important.

The purpose of this work was to study the electrothermal properties (that is, the ability to be heated under electrical load) of composites based on high-density polyethylene and carbon fillers (carbon black, short carbon fibers, and their mixtures), which form a segregated structure in the volume of the polymer matrix. The possible use of such a material for welding polymers is also shown.

## 2. Materials and Methods

### 2.1. Materials

Composites were molded using high-density polyethylene (HDPE) in the form of a fine powder with particle size *D* = 100–150 µm and density *ρ* = 0.95 g/cm^3^. The melting point was *T_m_* = 120 °C, melt flow index MFI = 1.15 g/10 min (Russian standard GOST 16338-85). As a conductive filler carbon black (CB) ENSACO^®^ 250 G, density *ρ* = 1.7 g/cm^3^ and chopped carbon fiber (CF) with particle size *d*~10 µm, *l* = 200–300 µm and density *ρ* = 1.9 g/cm^3^ (Russian standard GOST R 57407-2017) were used. The content of CB and CF in the composites was 8% by volume. Additionally, hybrid mixed filler CB/CF was processed at a concentration of 4 vol.% CB + 4 vol.% CF.

### 2.2. Sample Preparation

To obtain a segregated structure, the samples were hot-pressed. In the first stage, a homogeneous mechanical mixture was obtained by mixing HDPE and carbon filler powders. The filler was distributed over the surface of the polymer particles by thoroughly mixing the components in a mortar to obtain a layer of filler that covers the surface of the polymer grains since the particle size of the polymer *D* and filler *d* differed significantly (*D >> d*). During hot pressing, this structure was maintained, the polymer particles were deformed and joined into a continuous polymer matrix, while the filler particles were located at the boundaries between the polymer grains, forming a continuous conducting framework within the polymer matrix. In such a way, the so-called segregated structure of the conductive phase in the composite is formed [13].

In the second stage, the obtained mechanical mixture was placed in the steel closed mold heated up to 140 °C. The mixture was compacted for 5 min under a temperature of 140 °C and a pressure of 20 MPa. The samples were pressed in the form of discs with a diameter of 30 mm and a thickness of 1.5 mm. Previously, electrodes from the bonding wire were introduced into the mold, which was placed one opposite the other near the walls of the mold, thus the volume of the sample contained two built-in electrodes. Pressed samples were cooled in the mold under pressure to room temperature, using air cooling, after which they were removed from the mold.

**Figure 1:**
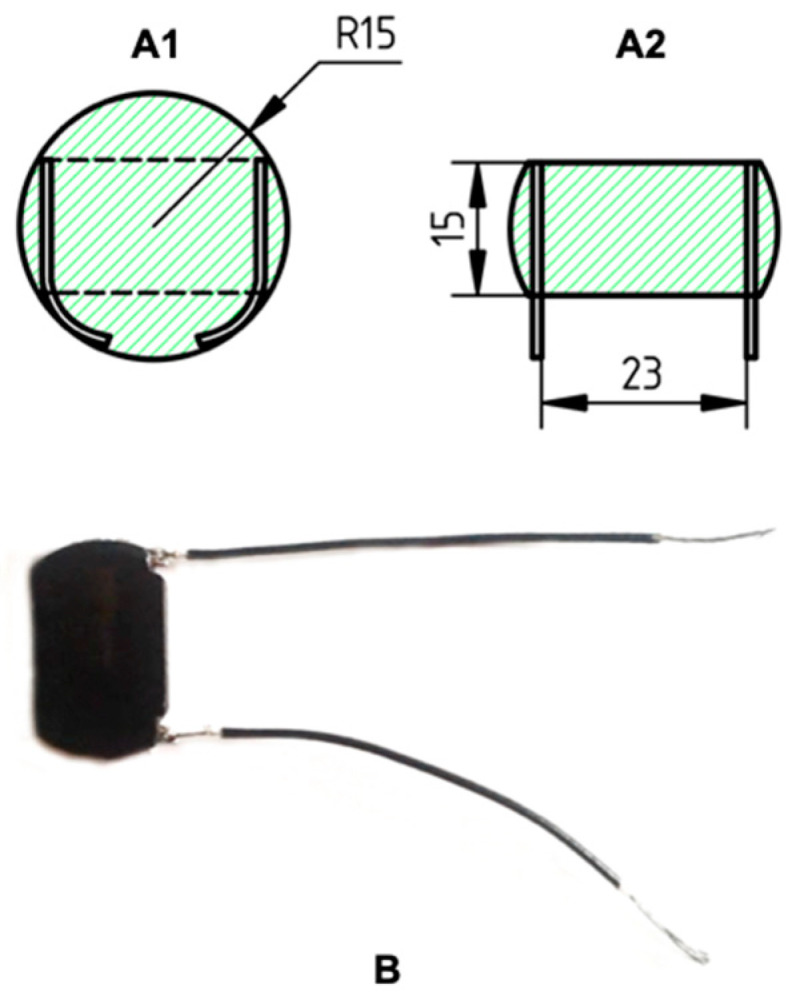
(**A1**) Sketch of the sample after hot pressing with build-in wires; (**A2**) cut sample with a thickness of 1.5 mm and ready-to-solder electrodes; (**B**) the sample of the composite prepared for electrothermal measurements.

For electrothermal studies, samples of the same size were cut from the disks in the form of a strip 15 mm wide in such a way that the built-in electrodes remained on the uncut ends of the strip, which is shown in Figure 1A1,A2. Wires were connected to the electrodes, through which voltage was applied to the sample. The image of the sample is given in Figure 1B. The processed composites, depending on the type of filler, were designated as HDPE-CB, HDPE-CF, and HDPE-CB/CF.

### 2.3. Measurement of Electrical and Thermal Characteristics

The electrical and thermal characteristics of the composites were studied by applying an alternating voltage to the sample in the range of 0–20 V from an alternating current source, that allows a maximum current of 2A. The voltage U (V) and the current I (A) in the circuit were recorded by multimeters UNI-T UT71B. The temperature T (°C) was measured by a thermocouple (type K) placed on the surface of the sample. A certain value of voltage was applied to the sample and the change in temperature and current over time was recorded through it, reaching a constant equilibrium temperature *T_e_* in a certain period.

## 3. Results and Discussion

### 3.1. Structure of Composites

**Figure 2:**
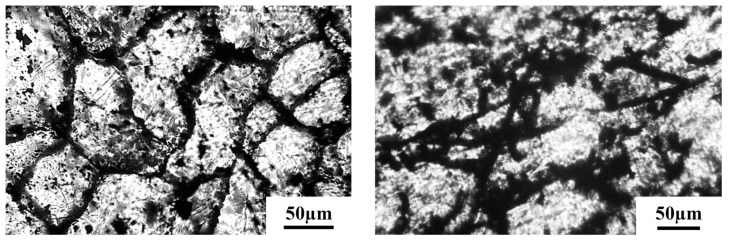
Optical microscopy of the segregated structure of HDPE-CB (**left**) and HDPE-CF (**right**) composites with a filler content of 4 vol.%.

The segregated structure of HDPE-CB and HDPE-CF composites is shown in Figure 2. As can be seen, the carbon fillers, CB and CF, were localized at the intergrain boundary, forming a conductive framework in the polymer matrix. In such a structure, the local concentration of filler particles in the grain boundaries was high and governs the electrophysical characteristics of the composite, such as electrical conductivity and thermal conductivity [39]. Filler particles form ohmic local contacts and form a percolation cluster, providing charge transfer at a low overall filler concentration.

The geometric model of the segregated system, proposed in [40] and developed in computer simulation [41], relates the value of the percolation threshold (that is, the appearance of electrical conductivity in the composite) with such parameters as the size of the polymer particles *D*, the filler *d*, and the number of filler layers *n* on the surface of the polymer grains, which determines the wall thickness of the conductive framework. According to the model, the concentration of filler particles in the walls of the framework is close to the maximum, which provides high conductivity with low overall filler content. Increasing the filler content leads to a thickening of the framework wall, which can be estimated from the equations given in [39]. This structure is extremely efficient in generating Joule heat when current flows through the framework because almost all of the filler particles are in contact with each other and conduct electrical current. For comparison, in the case of a random distribution of filler particles in the polymer matrix, only a small part of them constitutes a conducting cluster [42].

### 3.2. Kinetic Temperature Dependence of Composites

**Figure 3:**
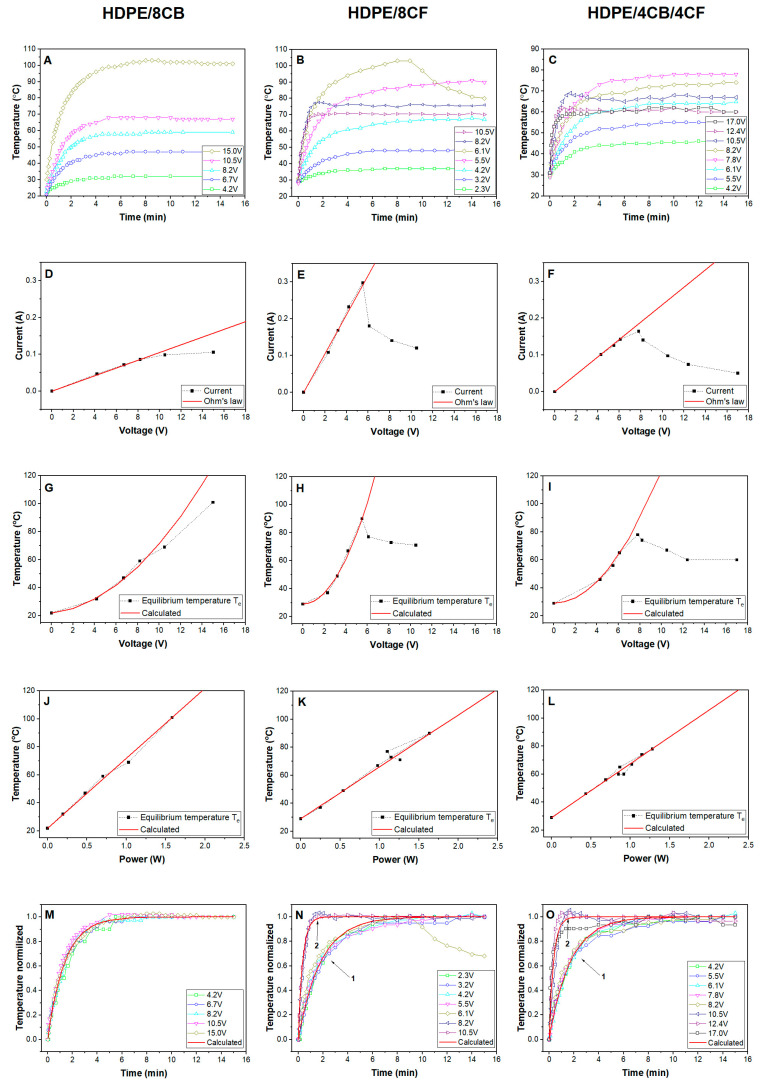
Electrothermal parameters of composites filled with carbon black (**A**,**D**,**G**,**J**,**M**), carbon fibers (**B**,**E**,**H**,**K**,**N**), and hybrid filler CB/CF (**C**,**F**,**I**,**L**,**O**).

The temperature kinetic curves for different values of the voltage that was applied to the HDPE-CB, HDPE-CF, and HDPE-CB/CF samples are shown in Figure 3A–C. As it can be seen, at first there was a rapid increase in temperature above the initial temperature T_0_, but after a certain time of keeping the sample under voltage, the equilibrium temperature *T_e_* is established. Its value is determined by the balance between Joule heat, which is released inside the sample due to the flow of current through the conductive filler, and heat dissipation by the sample into the environment (through radiation and convection [7]). As the voltage applied to the sample grew, the value of the equilibrium temperature *T_e_* increased. The value and behavior of the equilibrium temperature depend on many factors and are discussed below. Similar kinetic dependences were studied in works [7,23,26,43] for systems based on epoxy resin and graphene (Gr) [7], carbon nanotubes (CNT) [43], Gr/CNT mixture [26] and for a system based on polyethylene and CNT [23].

### 3.3. Electrical Properties of Composites

The relationship between voltage and current (current–voltage characteristics) is shown in Figure 3D–F. As can be seen, this dependence was linear in the interval from zero to a certain value of the voltage, exceeding which the value of the current decreased. This is a result of the PTC effect (positive temperature coefficient of resistance) and is discussed later. This effect is related to the behavior of the kinetic temperature curves at different values of the applied voltage to the sample. The linearity of this characteristic in areas below the appearance of the PTC effect indicates that the conductive phase of the filler behaves as an ohmic resistor, i.e., the particles of the filler form a conductive framework due to direct contact. The slopes of the straight lines were different, and they determine the electrical conductivity of the composites. A comparison of these dependencies (Figure 3D–F) for the three composites showed the greatest slope for the HDPE-CF composite, smaller for HDPE-CB/CF, and still smaller for HDPE-CB, which means the highest conductance of the HDPE-CF composite and less of the HDPE-CB/CF and HDPE-CB composites. Accordingly, the resistance R of the composites had an inverse value of the conductance, that is, the value of the resistance R = U/I of the HDPE-CF composite was the smallest. The calculated resistance values for HDPE-CF, HDPE-CB/CF, and HDPE-CB composites were 18.9, 41.7, and 100 Ohm, respectively, and are given in Table 1.

The dependence of the equilibrium temperature *T_e_* on the applied voltage *U* is shown in Figure 3G–I. As can be seen, the temperature depends on the applied voltage nonlinearly. This dependence is described by a quadratic equation:

(1) $$T_{e}=T_{0}+a\cdot U^{2}$$

where *a* is the coefficient, and *T*_0_ is the initial temperature. The values of the coefficients *a* for all composites are shown in Table 1. From Figure 3G–I, the excellent coincidence of the calculation according to Equation (1) (solid line) can be seen with the experimental values (points). The value of coefficient *a* depends on the resistance of the sample; the lower the resistance of the sample, the higher the value of coefficient *a*. A smaller resistance determines a larger amount of current through the sample, and this, in turn, leads to the entry of a larger electric power into the conductive phase of the composite. Consequently, the release of a greater Joule heat at a certain voltage value is observed and, as a result, an increase in the equilibrium temperature *T_e_*. This observation explains the steeper dependence of *T_e_*~*U* for the HDPE-CF composite than for HDPE-CB/CF and HDPE-CB (which has the minimal value of the resistance *R* and, accordingly, the maximal value of coefficient *a*).

Figure 3J–L shows the dependence of the equilibrium temperature *T_e_* on the power *P* supplied to the sample for HDPE-CB, HDPE-CF, and HDPE-CB/CF composites. It can be seen that this dependence was linear in the entire range of power and temperature changes, and is described by the following equation:

(2) $$T_{e}=T_{0}+b\cdot P$$

where the coefficient *b* has different values for each composite, which are presented in Table 1. This value depends on the conditions of heat transfer from the filler to the polymer matrix and the level of heat exchange of the sample with the environment. The shape of the sample, its mass, i.e., thickness, and area, also affect the value of the coefficient *b*. For a solid sample, the value of *b* will be greater than for a film (at the same resistance values), since the heat exchange that lowers *T_e_* is higher in the latter case.

The highest value of *b* = 50 °C/W was observed for the HDPE-CB composite, and the lowest for the HDPE-CF composite, *b* = 37 °C/W. Since the dimensions of the samples were the same for all composites, and the measurement conditions also did not differ, the difference in coefficients *b* for the composites is due to the efficiency of the transfer of electric power into the volume of the polymer in the form of heat. Since the value of the specific surface area *S_s_* of carbon black significantly exceeds that of carbon fiber, due to the existence of a large interphase surface, heat transfer from the filler phase to the polymer is much more efficient. For most types of carbon black, the specific surface is up to 100 m^2^/g [44,45], although for some types it reaches 600 m^2^/g [46]; however, the existence of carbon black in composites in the form of agglomerates significantly reduces the interfacial area between the filler and the polymer matrix. For the fiber that was used, the value of *S_s_* = 4/*dρ* = 0.2 m^2^/g (*d* is the diameter of the fiber, *ρ* is its density), i.e., it is orders of magnitude smaller than for carbon black. The coefficient *b* is calculated as the slope of the linear dependence *T_e_*~*P* (Figure 3J–L) and can be considered an indicator of the efficiency of heating the composite due to Joule heat.

In the temperature region where the value of equilibrium temperature is reached, the amount of heat gained by the sample through electrical power is equal to the amount of heat lost through radiation and convection from the surface of the sample. Accordingly, in refs. [7,23,26,43], the parameter *h_rc_*, which reflects the state of temperature equilibrium, is given as

(3) $$h_{rc}=\frac{I\cdot U}{T_{e}-T_{0}}$$

where *I* is the value of the current when the equilibrium temperature is reached, and *U* is the voltage applied to the sample. The parameter *h_rc_* shows how high-temperature *T_e_* can be reached in the composite by the applied electric power *P* = *I*·*U*. The smaller the value of *h_rc_*, the higher the efficiency of converting electrical power into heat (since the value *h_rc_* = 1/*b*, the value of *b* is directly related to the heating efficiency). The *h_rc_* values achieved in this work are equal to 20–27 mW/°C. In refs. [7,23,26,43], much smaller values of this parameter are given: 2.2 mW/°C [7], 4.9 mW/°C [43], 9.1 mW/°C [23], and 15.7 mW/°C [26]. Such a large difference can be explained by the fact that in the cited works, nano-sized fillers, graphene, and carbon nanotubes, which have a huge specific surface area and, therefore, a large interfacial filler-polymer surface, through which Joule heat is transferred from the filler to the polymer. Both carbon black and, even more so, carbon fiber have a much smaller specific surface area and, in this sense, are less efficient.

In addition, the properties of the polymer matrix also influence the heating parameters. Among the works cited, the composite based on polyethylene and CNT shows the highest value of *h_rc_* = 15.7 mW/°C [26], while the composite based on epoxy resin and CNT has *h_rc_* = 4.9 mW/°C [43]. Obviously, the strong adhesion of the epoxy resin to the filler, compared to polyethylene, contributes to better thermal contacts, i.e., lower thermal resistance, between the polymer and the filler and increases the efficiency of heat transfer (which reduces the value of *h_rc_*).

### 3.4. Influence of the PTC Effect on the Pyroresistive Properties of Composites

In the HDPE-CF composite, when a voltage of *U* > 5.5 V is applied, an unusual behavior of temperature versus time is observed. When the temperature reaches 102 °C, the current through the sample suddenly drops and the temperature decreases (Figure 3B). This phenomenon can be explained by the manifestation of the PTC (positive temperature coefficient of resistance) effect, which is due to the increase in the resistance of the sample as the temperature increases [47,48,49]. When heated in the premelting region, a significant thermal expansion of the polymer matrix occurs, which leads to the destruction of contacts between adjacent particles of the conductive filler, and the resistance of the composite increases. In turn, as a result, the current through the sample decreases, and, therefore, the electric power *P* applied to the sample decreases, which leads to a drop in temperature.

It can be seen from the Figure 3B that the further increase of the applied voltage to *U* = 8.2 V and 10.5 V changed the appearance of the kinetic curve; an initial rapid increase in temperature was observed, and then the temperature reached the equilibrium values of 75 and 80 °C.

The influence of the PTC effect is reflected in the voltage–current characteristic (Figure 3E) and the dependence of *T_e_*~*U* (Figure 3H). As can be seen, when the critical voltage *U_c_* > 5.5 V was exceeded and when the PTC effect began to operate, the experimental points deviated from the theoretical curves, and they fell below the points that coincide with the experimental ones. This abrupt change indicates a strong increase in the resistance of the sample as a result of its heating. Thus, the value of the equilibrium temperature depends on the change in the resistance of the sample.

The pattern of the PTC effect in the composite with HDPE-CB/CF hybrid filler is similar to the HDPE-CF composite. When the critical value of the voltage *U_c_* > 6.1 V was exceeded, the experimental values of the current (Figure 3F) and temperature (Figure 3I) fell below the theoretical values due to the increase in resistance. When the voltage applied to the sample increased, the value of the equilibrium temperature gradually reached a constant value of *T_e_* = 60 °C (Figure 3I); consequently, the composite exhibits self-regulating properties.

Regarding the HDPE-CB composite, the influence of the PTC effect manifested itself differently. Starting from the critical voltage value (*U_c_* ≥ 8.2 V), the equilibrium temperature lagged behind the theoretical values (Figure 3G), although it did not fall, but slightly increased with increasing the voltage applied to the sample. Additionally, the current values did not fall below the achieved values and reached a constant value (Figure 3D) and no sharp changes were observed on the kinetic curves (Figure 3A). Hence, for this composite, the PTC effect appeared softer and did not have a sharp character. The reason for this may be the different structures of the conductive phase for carbon black and carbon fibers. The branched CB structure, formed from CB agglomerates, is quite flexible and weakly destroyed by the thermal expansion of the polymer matrix. The conductive structure consisting of carbon fibers is sensitive to the thermal expansion of the polymer because the loss of even a few contacts between long fibers excludes from conduction a rather large part of such a filler. As for the CB/CF hybrid filler, the obtained results showed its behavior as similar to that of individual carbon fibers, i.e., the conductivity in the hybrid filler is mainly provided by the CF phase.

The magnitude of the relative increase in resistance during heating Δ*R*/*R* allows for a quantitative comparison of the influence of the PTC effect on composites, where Δ*R* = *R_e_* − *R*; *R* is the initial resistance value that corresponds to the linear part of the current–voltage characteristic (presented in Table 1), while *R_e_* is the experimentally determined resistance of the sample at a certain voltage. For example, at a voltage of 15 V for the HDPE-CB composite the value of Δ*R*/*R* is equal to 43%, while for the HDPE-CB/CF composite at the same voltage, Δ*R*/*R* = 500%. Hence, the increase in electrical resistance during heating as a result of Joule heat generation is greater in composites containing carbon fibers, which leads to a more significant limitation of the equilibrium temperature in HDPE-CF and HDPE-CB/CF composites. In the HDPE-CB composite, an equilibrium temperature of 101 °C was reached, while in the HDPE-CF and HDPE-CB/CF composites it was 70 °C and 60 °C, respectively (Figure 3A–C). Thus, the self-regulating effect occurred at different temperatures depending on the reaction of the conductive phase to the thermal expansion of the polymer matrix.

It should be noted that the PTC effect takes place in crystalline polymers, in which significant changes in the supramolecular structure occur in the premelting temperature region, leading to a strong expansion of the polymer volume [49], which, in turn, destroys the conductive network of the filler. Amorphous polymers, such as epoxy networks, are not affected by the PTC effect; therefore, the studies [7,26,43] do not observe such processes and show [50] dependence on the nature of the filler and its particles contacts; meanwhile, it is hard to evaluate the influence of each.

The dependence of the equilibrium temperature on the power shown in Figure 3J–L had a linear character and obeyed Equation (2) for all voltage values, from 2.3 to 17.0 V. This indicates that the conversion of electric power consumed by the conductive phase into heat flow in the polymer matrix occurs with a similar behavior in all cases, both for low and high voltage and independently of the influence of the PTC effect. The value of the equilibrium temperature *T_e_* is determined by the amount of heat generated by the conductive phase, i.e., by the electric power, which consists of the value of the applied voltage and the equilibrium value of the current. The PTC effect increased the resistance of the composite and, accordingly, reduced the current through the sample. The consequence was a reduction of the electrical power and a decrease in the equilibrium temperature. This is the reason for the linear dependence of *T_e_*~*P* shown in Figure 3J–L, regardless of the presence or absence of the PTC effect.

### 3.5. The Influence of Voltage on the Kinetic Heating

Figure 3A–C shows the kinetic dependencies of the temperature of the composites during heating at different voltage values. It can be seen that the kinetic curves are similar and differ only in the value of the equilibrium temperature when different voltage values are applied to the sample. Therefore, the curves can be presented in a generalized form, using the normalized value of the temperature *T_n_*:

(4) $$T_{n}=\frac{T-T_{0}}{T_{e}-T_{0}}$$

where *T* is the current temperature, and *T*_0_ is the initial temperature. By normalizing each temperature curve, a generalized kinetic curve can be obtained for all values of the applied voltage. As can be seen from Figure 3M–O, the normalized experimental data of each composite fit well to a generalized curve.

In refs. [7,43], the kinetic curve is proposed to be described by the following equation:

(5) $$T_{n}=1-exp\left(-\frac{t}{t_{c}}\right)$$

where *t* is the exposure time of the sample under voltage, and *t_c_* is the characteristic time. According to [43], the last parameter indicates the speed of the temperature response to the applied voltage. Equation (5) can be written in the following form:

(6) $$T_{n}=1-exp\left(-kt\right)$$

where *k* is the rate constant of the sample heating process, which is the reciprocal of the characteristic time, *k* = 1/*t_c_*. The dependence of normalized temperature on time, calculated according to Equation (6), is shown in Figure 3M–O by solid lines. It can be seen that the theoretical curve coincides well with the experimental points at certain values of *k* (or *t_c_*), which are presented in Table 1. The values of *t_c_* for HDPE-CB, HDPE-CF, and HDPE-CB/CF composites are equal to 73.8, 109.2, and 101.4 s, respectively. The smaller the value of *t_c_* (or the larger the rate constant *k*), the faster the sample is heated and the equilibrium value is reached, when the generation of Joule heat is balanced by its exchange with the environment.

It should be noted that the rate of heating and reaching the equilibrium temperature *T_e_* depends on the shape of the sample, in films this process is fast, while the heating of a massive sample is much slower. According to the authors [7,43], in composites based on epoxy resin and graphene or carbon nanotubes, the values of *t_c_* are equal to 5.7 and 5.2 s, respectively, and in the polyethylene-carbon nanotube composite *t_c_* = 15.4 s [26]. This is much less than for the composites studied. However, in these works, the samples were thin films, for example, in [7], the film samples were 0.1 mm thick, compared to the 2 mm thick for our samples.

It is seen from Figure 3N,O that the set of curves is divided into two families for HDPE-CF and HDPE-CB/CF composites. For the HDPE-CF composite, the application of a voltage in the range *U* = 2.3–6.1 V produces a generalized curve 1 similar to that of the HDPE-CB composite, while the temperature data for higher voltages *U* = 8.2–10.5 V give another generalized curve 2. This effect indicates a change in the mechanism of conversion of electrical power into thermal energy. The characteristic time for generalized curve 1 is *t_c_*_1_ = 109.2 s, while for curve 2 this parameter is equal to *t_c_*_2_ = 21.6 s, which indicates an abnormally high rate of temperature rise at elevated voltages. As for the HDPE-CB/CF composite, a similar picture is observed. In the voltage interval 4.2–8.2 V, there is a generalized curve 1 with a value of *t_c_*_1_ = 101.4 s, while a higher voltage interval of 10.5–17 V leads to the formation of another generalized curve 2 with a characteristic time value of *t_c_*_2_ = 21.0 s. Accordingly, the rate constants *k* of the heating process (see Table 1) reflect these features of the generalized kinetic curves and their values differ by ~5 times for curves 1 and 2.

The anomalous behavior of HDPE-CF and HDPE-CB/CF composites at high voltages is probably explained by the electrical breakdown of thin polymer layers or poor contact between elements of the conductive phase of carbon fibers after applying a higher voltage. Since these elements of the conductive phase are quite large, such phenomena lead to a sharp increase in the current through the conductive framework of the filler, due to which a large amount of Joule heat is released. This leads to a rapid rise in temperature. On the other hand, it also causes local overheating of the polymer matrix near the conducting elements of the filler, which causes thermal expansion of the polymer and destruction of contacts between particles. The result of this is a decrease in the current through the conductive phase and a decrease in the power that generates heat, and, which leads to a decrease in the equilibrium temperature (Figure 3B,C). For the HDPE-CB composite, such an effect is absent due to a different structure of the conductive phase.

All three composites exhibit a self-regulating effect relative to the equilibrium temperature, which is reached when the voltage applied to the sample is increased. The highest equilibrium temperature of 101 °C belongs to the HDPE-CB composite at a voltage of 15 V, for the HDPE-CF composite the equilibrium temperature reaches 70 °C at 10.5 V, and for the HDPE-CB/CF composite an equilibrium temperature of 60 °C is established at a voltage of 12.4–17.0 V. By controllable formation of the conductive phase of the filler, it is possible to regulate this parameter within fairly wide limits for various applications of this type of composites.

Generally, according to the sum of the parameters, the HDPE-CB composite seems to be preferable for creating a heating element. As can be seen from Table 1, the values of the parameters *b* and *h* = 1*/b* showed a higher efficiency of converting electrical power into heat for this composite. In addition, the PTC effect is not as drastic for the HDPE-CB composite as for the HDPE-CF and HDPE-CB/CF composites, which makes it possible to achieve a higher heating temperature. From Figure 3M–O, it can be seen that the HDPE-CB composite exhibited a more uniform heating process as a function of applied voltage, in contrast to the HDPE-CF and HDPE-CB/CF composites, which exhibited two heating rate constants *k* (see Table 1) and, respectively, two types of kinetic curves depending on voltage. Thus, the HDPE-CB composite was chosen for the manufacture of the heating element.

### 3.6. Application

As mentioned above, polymer composites with pyroresistive properties can be used in various fields of technology; their possible areas of use are quite fully covered in the overview [1]. Nevertheless, it is worth mentioning another promising area of application of pyroresistive polymer composites, this is the welding of plastic parts with a heating element (HE) [51]. This type of welding, the so-called resistance welding or fusion bonding, is used to connect parts of complex geometry or polymers with a high melting point. In this case, a heating element in the form of a metal mesh is usually used [52,53]. When the current flows through the HE, it is heated by Joule heat and the surface layer of the polymer parts melts, parts which are then bonded together. Since the mesh remains in the welded joint, it deteriorates the mechanical properties of the final product. Therefore, the use of heating elements made of pyroresistive composites with the same polymer matrix as in the parts to be joined can ensure the uniformity of the welded joint and its strength [38,54].

**Figure 4:**
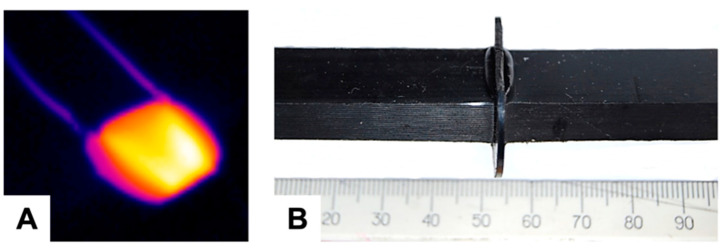
(**A**) Image of a heated heating element; (**B**) welded joint of HDPE parts with the HDPE-CB heating element.

The samples of the HDPE-CB composite shown in Figure 1B were used as a heating element, with the difference that the CB content was increased to 30 vol.%. Carbon black was chosen as an electrically conductive filler since CB is an affordable, cheap, and widely used conductive filler that provides a high level of electrical conductivity and stable electrical characteristics during product operation. The increase in the CB concentration in the heating element was provided because the CB content in the composite in the amount of 8 vol.% ensures that the composite is heated to 101 °C, which is not enough to form a welded joint. With a CB content of 30%, the composite can be heated up to 200–300 °C in the welding zone [38]. Figure 4A shows an image of a heating element in a heated state, obtained with the use of an infrared thermal imager.

Figure 4B shows an HDPE butt weld formed with the HDPE-CB heating element. The presence of burr in the welding zone indicates that the HE provides the necessary heating and melting of the surface of the parts to be welded. A detailed study of welded joints of polyethylene parts obtained using polyethylene-based pyroresistive heating elements showed that the mechanical strength of the welded joint depends on the welding regime [38]. The optimal regime, determined by the electric power supplied to the HE and the welding time, ensures the strength of the welded joint at the level of the strength of the parts being welded. Thus, resistance welding using a heating element made of a pyroresistive material of the same nature as the parts to be welded gives high-quality welding and is a very promising method for use in various industries.

## 4. Conclusions

Kinetic patterns of heating of conductive composites based on high-density polyethylene and carbon fillers, namely carbon black, carbon fibers, and their mixtures, were studied. It is shown that the kinetic curves are well described by an exponential dependence with different rate constants of the heating process. It was revealed that composites containing carbon fiber (HDPE-CF and HDPE-CF/CB) have two types of kinetic curves, with normal and abnormal heating rates, for which the rate constants differ by ~5 times. After a fairly short heating time, the equilibrium temperature *T_e_* was established in the sample.

Electrothermal processes during the heating of composites with different electric power, which generates the release of Joule heat in the composite, were also studied. It was shown that the equilibrium temperature depends on the applied voltage according to the quadratic law whereas there is a linear relationship between the equilibrium temperature and electric power.

When the applied voltage exceeds a certain value in the HDPE-CB composite, the equilibrium temperature slowed down its growth while in the HDPE-CF and HDPE-CB/CF composites *T_e_* decreased due to the presence of the PTC (positive temperature coefficient) effect. It consists of the thermal expansion of the polymer matrix, which partially destroys the conductive chains of the filler, which increases the electrical resistance and, in turn, reduces the electrical power that generates Joule heat. The conductive phase formed from carbon fiber or a mixture of CF and CB is more sensitive to the presence of the PTC effect than carbon black.

Due to the PTC effect, all three composites exhibited a self-regulating effect relative to the equilibrium temperature, which was reached when the voltage applied to the sample was increased. In the HDPE-CB composite, an equilibrium temperature of 101 °C was established at a voltage of 15 V, for the HDPE-CF composite, the equilibrium temperature reached 70 °C at 10.5 V, and for the HDPE-CB/CF composite, an equilibrium temperature of 60 °C was established at a voltage of 12.4–17.0 V. By the controllable formation of the conductive phase of the filler, it is possible to adjust this parameter within fairly wide limits for various applications of this type of composites.

The use of HDPE-CB composite to create HE for resistance welding was demonstrated and the welded joint of HDPE parts obtained using HE was shown. A possible application of such pyroresistive composites is resistance welding of plastics using a heating element made of a pyroresistive material of the same nature as the parts to be welded. This method gives a high quality of the welded joint and is very promising for use in various industries.

## Figures and Tables

**Table 1:** Parameters of Equations (1)–(3), (5), and (6).

		8CB	8CF	4CB/4CF
1*/R*	[S]	10 × 10^−3^	53 × 10^−3^	24 × 10^−3^
*R*	[Ohm]	100	18.9	41.7
*a*	[°C/V^2^]	0.46	2.0	0.94
*b*	[°C/W]	50.0	37.0	38.5
*h_rc_* = 1*/b*	[mW/°C]	20	27	26
*t_c_*	[s]	73.8	21.6/109.2	21.0/101.4
*k* = 1*/t_c_*	[s^−1^]	136 × 10^−3^	46.3/9.2 × 10^−3^	47.6/9.9 × 10^−3^

## Data Availability

Not applicable.
